# Progress on Understanding Transcriptional Regulation of Chloroplast Development in Fleshy Fruit

**DOI:** 10.3390/ijms21186951

**Published:** 2020-09-22

**Authors:** Ting Jia, Yuting Cheng, Imran Khan, Xuan Zhao, Tongyu Gu, Xueyun Hu

**Affiliations:** 1 Key Laboratory of Plant Functional Genomics of the Ministry of Education, Yangzhou University, Yangzhou 225009, China; tingj2012@yzu.edu.cn; 2 Joint International Research Laboratory of Agriculture and Agri-Product Safety of the Ministry of Education of China, Yangzhou University, Yangzhou 225009, China; 3 College of Bioscience and Biotechnology, Yangzhou University, Yangzhou 225009, China; dx120200180@yzu.edu.cn (Y.C.); dh18006@yzu.edu.cn (I.K.); mx120190786@yzu.edu.cn (X.Z.); mz120191171@yzu.edu.cn (T.G.)

**Keywords:** fleshy fruit, chloroplast development, chromoplast, nutritional quality, pigment

## Abstract

Edible fleshy fruits are important food sources in the human diet. Their yield and nutritional quality have long been considered as breeding targets for improvement. Various developing fleshy fruits with functional chloroplasts are capable of photosynthesis and contribute to fruit photosynthate, leading to the accumulation of metabolites associated with nutritional quality in ripe fruit. Although tomato high-pigment mutants with dark-green fruits have been isolated for more than 100 years, our understanding of the mechanism of chloroplast development in fleshy fruit remain poor. During the past few years, several transcription factors that regulate chloroplast development in fleshy fruit were identified through map-based cloning. In addition, substantial progress has been made in elucidating the mechanisms that how these transcription factors regulate chloroplast development. This review provides a summary and update on this progress, with a framework for further investigations of the multifaceted and hierarchical regulation of chloroplast development in fleshy fruit.

## 1. Introduction

Chloroplasts are essential organelles for converting light energy into chemical energy in plant and algal cells. They are not only vital for photosynthesis but also play a pivotal role in plant primary and secondary metabolism, such as the metabolism of amino acids, fatty acids, and phytohormones [1,2]. Given the importance of plant products to human survival, photosynthesis and the biogenesis of chloroplasts have received intensive investigation. In land plants, chloroplasts develop from proplastids that are present in developing meristems, however, chloroplast development are throughout all stages of plant growth [3,4]. For example, within a given *Arabidopsis* leaf, chloroplast developmental gradients can be observed from the base to the tip and from the margin to the midrib [3,5]. In this review, we also consider that chloroplast division is an integral part of normal chloroplast development, as it was suggested by Pyke [6]. To optimize photosynthesis to varying light regimes, plants have developed a complex and highly regulated chloroplast development process, which may be governed by light signaling, retrograde chloroplast to nucleus signaling, cell-specific chloroplast development, and photosynthetic acclimation to the light environment, as reviewed by Bapat et al. [7], Pfalz and Pfannschmidt [8] and Pogson et al. [3].

**Figure 1:**
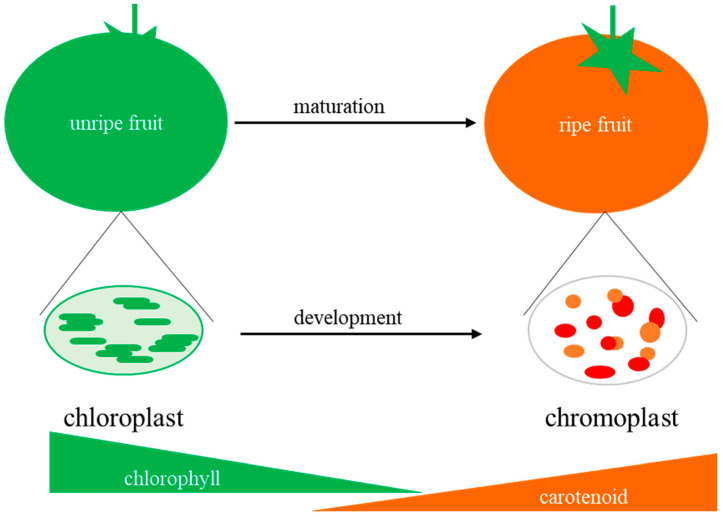
Chloroplast to chromoplast transition during fleshy fruit maturation. During fleshy fruit ripening, chloroplasts undergo changes to form chromoplasts, such as chlorophyll degradation and accumulation of metabolites (such as carotenoids) that contribute to nutritional quality of ripe fruit.

Leaves are the major site of photosynthesis for most plants, however, developing fleshy fruits are capable of photosynthesis, and it is estimated that up to 20% of the total fruit carbohydrate comes from the photosynthesis of immature fleshy fruits in tomato [9], the model plant for fleshy fruits. In addition to photosynthesis, during fruit maturation, chloroplasts develop into chromoplasts, synthesizing nutritional metabolites, such as carotenoids (Figure 1) [10]. The transition from chloroplast to chromoplast is often happened in flowers and fruits, but only a few plant species differentiate chromoplasts in leaves [11,12]. During chloroplast to chromoplast transition, besides the remodeling of the internal plastid structures, the most prominent changes are a degradation of photosynthetic competence and over-accumulation of carotenoid pigments, as it was well reviewed by Egea et al. [13]. Therefore, the manipulation of chloroplast to chromoplast differentiation in plants appears as a very promising strategy for improving the nutritional and health benefits of crops [14,15]. On the other hand, it has been demonstrated that tomato fruits with more active chloroplasts at the premature stage will develop more active chromoplasts at the mature stage [16]. Therefore, enhancing fruit chloroplast development can result in higher nutritional value in mature fleshy fruit [17,18,19].

In general, chloroplast development in fruits shares similar regulatory mechanisms with those found in leaves [17,20]. However, it was discovered that some transcription factors have special contribution on the regulation of fruit chloroplast development, while they only have little effect on leaf chloroplast development [17,21,22]. In the past few years, understanding the molecular mechanism underlying the fine manipulation of chloroplast development in fleshy fruits has been intensively investigated. In this review, we provide an updated summary of the progress made in elucidating the transcriptional regulation of chloroplast development in fleshy fruits, and highlight major knowledge gaps for future study. Finally, strategies for improving fruit nutritional quality through manipulation of chloroplast development in fruits are proposed.

## 2. Key Transcription Factors that Regulate Chloroplast Development in Fleshy Fruits

### 2.1. GLK2 Is a Well-Studied Master Regulator of Chloroplast Development in Fleshy Fruits

Map-based cloning of the *uniform ripening* (*u*) mutation revealed that *U* encodes a *Golden 2-like* MYB transcription factor (GLK2), which is a positive regulator of chloroplast development and pigment accumulation in tomato (*Solanum lycopersicum*) fruit [17,23]. Two *GLK* genes are expressed in tomato, and the *SlGLK2* mRNA predominates in fruit. Increasing the expression of *SlGLK2* increases chloroplast number and size, producing homogeneously dark-green fruits with enhanced chlorophyll content [17].

Previously, it was reported that GLK transcription factors are conserved in plants and positively regulate chloroplast development in several land plants [24]. In *Arabidopsis*, there are also two *GLK* genes (*AtGLK1* and *AtGLK2*), which function redundantly to regulate chloroplast biogenesis in different plant organs. For example, in siliques, *AtGLK2* transcripts predominate, while *AtGLK1* transcripts do not accumulate; therefore, *AtGLK2* knockout mutants exhibit pale-green siliques [24]. By targeting the promoters of a suite of genes associated with photosystem biogenesis and chlorophyll biosynthesis, *Arabidopsis* GLKs coordinate the transcription of these genes, suggesting that GLKs function in positively regulating chloroplast development [25]. Constitutive overexpression of *AtGLK1* and *AtGLK2* in tomato increased both the number and size of green fruit chloroplasts and promoted the accumulation and development of grana thylakoids, resulting in increased soluble solids and carotenoids in ripe fruit, for better nutritional quality, while no obvious alterations of leaf chlorophyll or chloroplasts were observed [17]. In pepper (*Capsicum annuum* L.), it was reported that *CaGLK2* is a major quantitative trait locus controlling the natural variation in chlorophyll content and chloroplast compartment size in immature fruit [26]. Heterologous expression of the kiwifruit (*Actinidia chinensis*) GLK2 homologue in tomato resulted in dark-green immature fruit with increased chloroplast number and size (with enhanced thickness of thylakoid grana stacks) and ripened fruit with elevated contents of carotenoids and sugars [27]. Thus, GLK2 is a widely conserved master transcription factors, which possess specific contribution on the regulation of chloroplast development in fleshy fruits.

### 2.2. APRR2-Like Transcription Factors Regulate Chloroplast Development in Fleshy Fruits

Another transcription factor, ARABIDOPSIS PSEUDO RESPONSE REGULATOR2-LIKE (APRR2-Like) was identified by neural network inference analysis in tomato [18]. The sequence of SlAPRR2-Like has strong homology to that of APRR2, related to but distinct from SlGLK2 [24]. *SlAPRR2-Like* was upregulated at the breaker stage in wild-type tomato fruits, whilst *SlAPRR2-Like* overexpressing plants showed increased plastid number and area, higher chlorophyll content in green unripe fruits and elevated carotenoid content in red ripe fruits. However, although the level of *APRR2-Like* expression in the leaves of the transgenic lines was substantially higher than in the wild-type, the leaf of both transgenic and wild-type had similar total chlorophyll levels. Unlike *SlGLK2*, *SlAPRR2-Like* was suggested to play a role in the ripening process, but the underlying mechanism requires further investigation [18]. In the same report, a putative orthologue of the *SlAPRR2-Like* gene in sweet pepper (*Capsicum annuum* L.) was found to be associated with pigment accumulation in fruit tissues [18]. Recently, map-based cloning identified that a single-base insertion in the *APRR2-Like* gene leading to a premature stop codon is responsible for the white immature fruit colour in cucumber (*Cucumis sativus* L.) [28]. More recently, it was reported that the *APRR2* gene regulates pigment accumulation in melon and watermelon [29]. Taken together, these studies indicate that the *APRR2-Like* gene has a conserved function in promoting fruit pigment accumulation and chloroplast development in flowering plants. Thus far, the underlying molecular mechanism of action of *APRR2* is unclear.

Since GLK2 is not required for the elevated pigment phenotype of *APRR2-Like* overexpression lines and the expression of *GLK1* was unchanged in *APRR2-Like* overexpressing plants [18,30], it was suggested that APRR2-Like is unlikely to regulate chloroplast development by mediating the expression of *GLKs*. On the other hand, *APRR2-Like* gene expression is not changed in *GLK2* overexpressing plants [17]. Although the relationship between *GLKs* and *APRR2-Like* needs further investigation, it was suggested that APRR2-Like and GLK2 act independently as key transcription factors to directly activate genes involved in fruit chloroplast development [21].

### 2.3. TKN2 and TKN4 Regulate the Expression of GLK2 and APRR2

*TKN2* and *TKN4*, which belong to the class I *KNOTTED-LIKE HOMEODOMAIN* (*KNOX*) gene family, were identified to play specific roles in chloroplast development in fruit but not in leaves [21]. Map-based cloning identified a point mutation in *TKN4* response to a mutant lacks the green shoulder of developing fruit. The development of chloroplast is impaired, with a reduction in size and in the number of thylakoids per granum in the fruit shoulder of *TKN4* unexpressed mutant [21] A dominant gain-of-function mutation that results in ectopic expression of *TKN2* is characterized by increased chloroplast number, higher chlorophyll content and larger size of chloroplast in tomato fruits. It is striking that these phenotypes resemble over-expression lines of *SlGLK2* and *SlAPRR2-Like*. Further investigation in tomato showed that TKN2 promotes chloroplast development in fleshy fruit by regulating both *SlGLK2* and *SlAPRR2-Like* expression, whereas TKN4 only regulates the expression of *SlGLK2* [21]. It was also found that TKN2 and TKN4 regulate the latitudinal gradient of expression of *GLK2* and *APRR2-Like* across developing tomato fruit, correlating with a gradient in chloroplast development [21].

### 2.4. Other Transcription Factors Regulate Chloroplast Development in Fruit

#### 2.4.1. BELs Negatively Regulate Chloroplast Development and Chlorophyll Synthesis in Tomato Fruit Directly and Indirectly

More recently, seven homeodomain-containing transcription factors with specific ripening-associated expression patterns were investigated, and three BEL1-like (BELL) members showed fruit-specific expression in tomato [22]. BEL1-LIKE HOMEODOMAIN11 (SlBEL11) was shown to regulate chloroplast development and chlorophyll synthesis in tomato fruits. When *SlBEL11* expression was reduced by RNAi, chlorophyll levels, chloroplast numbers per cell and the number of thylakoids per chloroplast were significantly increased in unripe tomato fruits, and 48 genes involved in chlorophyll biosynthesis, chloroplast development and photosynthesis were upregulated. Among the upregulated genes in *SlBEL11*-RNAi plants, 22 appear to be potential targets of SlBEL11 [22]. Furthermore, strong evidence showed that SlBEL11 could repress the expression of *TKN2*, chlorophyll *a*/*b* binding protein (*CAB*) and chlorophyll biosynthesis-related genes encoding for protochlorophyllide reductase (*POR*) by directly binding their promoters in green fruits but had no obvious effect in leaves [22]. Moreover, it was found that *DEETIOLATED1* (*DET1*), a negative regulator of photomorphogenesis and chloroplast development in fleshy fruit [31,32,33], was also repressed in *SlBEL11*-RNAi plants [22], indicating that SlBEL11 may also regulate chloroplast development through DET1. Most recently, it was reported that slightly darker-green tomato fruit with increased chlorophyll content, the number of thylakoids per granum of chloroplasts, starch, fructose and glucose resulted from downregulation of *BEL1-LIKE HOMEODOMAIN4* (*SlBL4*). Similar to SlBEL11, SlBL4 also directly inhibits some genes that encode enzymes catalyzing chlorophyll biosynthesis and *SlTKN2* [34]. Therefore, BELs play hierarchical roles in fruit chloroplast development.

#### 2.4.2. LOL1 Controls Chloroplast Size in a Fruit-Specific Manner

QTL map-based cloning identified a zinc-finger transcription factor CcLOL1 (LSD ONE LIKE1; CcLOL1) that regulates chlorophyll content by controlling chloroplast size in a fruit-specific manner in pepper (*Capsicum chinense*) [35]. SlLOL1 retains a similar function in tomato fruit [35], knockout of tomato *SlLOL1* results in light-green immature fruit with reduced chloroplast size and number. *LOL1* was initially identified as a positive regulator of programmed cell death in *Arabidopsis* [36]. Its rice (*Oryza sativa* L.) homolog, termed *OsLSD1*, also control programmed cell death. In addition, overexpressing *OsLSD1* resulted in an increase of chlorophyll content in the shoot [37]. However, knockout of *CcLOL1* or tomato *SlLOL1* had no significant effect on leaf chlorophyll content [35]. The mechanism by which LOL1 regulates chloroplast development in fleshy fruit is not well known, only transcriptome analysis revealed that genes involved in light harvesting, chlorophyll biosynthesis and *Rbc* genes (encoding ribulose bisphosphate carboxylase subunits) were significantly reduced in *CcLOL1* mutated bulk, suggesting the possible involvement of LOL1 in regulation of carbon fixation in the fruit [35].

## 3. Light Signaling Pathway Involved in the Transcriptional Regulation of Chloroplast Development in Fleshy Fruits

In 1917, five photomorphogenic mutants carrying monogenic recessive *high-pigment* (*hp-1*, *hp-1^W^*, *hp-2*, *hp-2^j^*, and *hp-2^dg^*) mutations were reported [38]. These mutants were characterized by exaggerated light responsiveness and intense fruit pigmentation in tomato, as extensively reviewed [39]. Characteristic phenotypes include dark-green fruits, resulting from increased plastid compartment size, increased chloroplast number, and higher chlorophyll content at pre-ripening stages [31,40,41,42]. Later, *hp-1* and *hp-1^W^* were mapped to the *HP-1 gene*, which is located on chromosome 2 [43], while *hp-2*, *hp-2^j^*, and *hp-2^dg^* were found to be allelic and were mapped to the *HP-2* gene located on chromosome 1 [44]. Furthermore, cloning of the *HP-1* gene revealed that it is the tomato homologue of the *Arabidopsis thaliana* gene encoding UV-DAMAGED DNA BINDING PROTEIN1 (DDB1) [39,41].

The *HP-2* gene encodes the tomato homologue of *Arabidopsis* DEETIOLATED1 (DET1) [31,32], a negative regulator of photomorphogenesis [33]. In fact, complex purification experiments has shown that DET1 interacts with the DDB1 protein [45]. They form a complex with Cullin4 (CUL4), a ubiquitin-conjugating E3 ligase, targeting proteins for proteolysis in *Arabidopsis* [46,47]. Therefore, it was suggested that the CUL4-DDB1-DET1 complex participates in chloroplast development and secondary metabolism in tomato fruits [48]. Research results showed that constitutive downregulation of *SlCUL4* resulted in greater plastid number and higher chlorophyll in unripe fruits and higher carotenoid in ripe fruits, similar to the phenotypes of *hp1* mutant and *SlDDB1*-RNAi plants [48]. Later, it was uncovered that SlGLK2 is a substrate of the CUL4-DDB1-DET1 ubiquitin ligase complex for proteasomal degradation [49].

The CUL4-DDB1-DET1 complex plays a vital role in regulating plant photomorphogenesis, suggesting that the light signal transduction pathway acts an important role in regulating chloroplast development in immature fleshy fruits. To test this hypothesis, *LeHY5*, the tomato homologue of *ELONGATED HYPOCOTYL 5* (*HY5*) and *LeCOP1LIKE*, the tomato homologue of *CONSTITUTIVELY PHOTOMORPHOGENIC 1* (*COP1*), which encode positive and negative regulators of light signaling, respectively, were knocked down by RNAi in tomato [41]. *LeHY5*-RNAi plants showed lighter green leaves and lighter green immature fruits with reduction of thylakoid organization and abundance in comparison to wild-type. In contrast, *LeCOP1LIKE*-RNAi plants exhibited significantly higher chlorophyll content in the leaf and immature fruit [41]. Furthermore, it was discovered that ripe fruits from *hp-1*, *hp-2* and *LeCOP1LIKE*-RNAi lines had increased total carotenoid levels, whereas ripe fruits of *LeHY5*-deficient plants possessed less carotenoid than the wild-type [41,42,43,50]. It was reported that COP1 and SPA (SUPPRESSOR OF PHYA) form a complex together with DDB1-CUL4, targeting HY5 for ubiquitination and degradation [51]. HY5, a master regulator that modulates light-regulated seedling photomorphogenic development, binds to the promoters of almost one-third of *Arabidopsis* genes [52]. HY5 directly regulates photosynthesis-related genes, such as *CAB1* and *RbcS1A* (encoding Rbc small chain 1A) [52]. It was also demonstrated that HY5 induces the expression of many genes that are related to root greening by binding to G-box elements (CACGTG) in their promoters [53]. The primary G-box elements for HY5 binding are also highly represented in the promoter of *GLK2*, but intriguingly not *GLK1*; therefore, *GLK2* is also a putative target of HY5 [25,52]. Moreover, it was found that overexpressing *GLKs* can only partly induce root greening in the absence of HY5, suggesting that HY5 plays an important role in *GLK* functions in the root [53]. However, *GLK2* has not yet been demonstrated to be a direct transcriptional target of HY5, and thus the relationship between HY5 and *GLK2* requires further investigation.

Recently, researchers discovered a protein containing a methyl-CpG-binding domain (MBD) in tomato, which was named SlMBD5, is a close homologue of *Arabidopsis* AtMBD5. The relative expression level of *SlMBD5* is remarkably high in fruit, while it is low in leaves. Overexpressing *SlMBD5* resulted in dark-green leaves and immature fruits [54]. As in the dark-green fruits of *hp1*, the number and size of chloroplasts were increased, the chloroplast total area per cell was greater in the outer pericarp of immature green fruits, and levels of lycopene and β-carotenoids were higher in the ripe fruits of *SlMBD5*-overexpressing lines in comparison to wild-type [54]. Repressing *SlMBD5* by RNAi did not obviously change the phenotype from wild-type, suggesting that SlMBD5 is dispensable for fruit chloroplast development. Further analysis showed that SlMBD5 physically interacts with SlDDB1, and it also interacts with CUL4, DET1, RING-BOX 1a(RBX1a) and RBX1b, supporting the idea that SlMBD5 is functionally linked to the known CUL4-DDB1-based complex [54]. The expression of *SlGLK2* was increased in the immature fruits of *SlMBD5*-overexpressing lines and *hp1* mutants, suggesting that the physical interaction between SlMBD5 and DDB1-based complex mediates the transcriptional activities of downstream genes, although the mechanism is still largely unknown [54]. Furthermore, yeast two-hybrid and co-immunoprecipitation assays suggested that SlMBD5 also interacts with the SlGLK2 protein in vivo [54]. SlMBD5 may affect SlGLK2 degradation through the CUL4-DDB1-DET1 ubiquitin ligase complex, which plays a vital role in SlGLK2 degradation by the 26 S proteasome [49].

Transcript levels of *SlGLK2* were elevated in *SlMBD5*-overexpressing lines, and the expression of *SlAPRR2-Like* as well as *SlGLK2* was considerably enhanced in the *hp1* mutant, indicating that the DDB1 signaling pathway plays roles upstream of *SlAPRR2-Like* and *SlGLK2* to regulate chloroplast development [21,55]. The expression of *TKN2* and *TKN4* was also significantly enhanced in the *hp-1* mutant. Therefore, it was suggested that TKN2 and TKN4 work downstream of DDB1 and upstream of GLK2 and APRR2 to regulate fruit chloroplast development in tomato [21]. Further investigation is needed to determine whether TKN2 and TKN4 bind directly to the regulatory promoter regions of *SlGLK2* and *SlAPRR2-Like*, and to understand how DDB1 regulates the expression of *TKN2* and *TKN4* [21].

UV (Ultraviolet) RESISTANCE LOCUS8 (UVR8), the unique UV-B photoreceptor in the UV-B signaling pathway, usually exists as a homodimer, but it is easily converted to monomers in response to UV-B irradiation [56]. Monomerized UVR8 interacts with the COP1-SPA protein complex to form the UVR8-COP1-SPA complex, which negatively affects COP1-SPA1 activity while stabilizing and promoting its HY5 activity [57,58,59]. Thus the UVR8-COP1-SPA complex can control fruit chloroplast development via HY5. In tomato, *SlUVR8* is required for the expression of UV-B-induced *SlGLK2* in fruits and leaves. Moreover, it was suggested that SlUVR8 also plays a role in promoting chloroplast development by enhancing the accumulation of SlGLK2 protein under UV-B illumination through post-translational regulation [59]. In *SlUVR8*-overexpressing plants, higher *SlUVR8* levels resulted in a larger plastid area per cell and increased chlorophyll content in immature green fruits. In contrast, decreasing the expression of *SlUVR8* resulted in smaller plastid areas per cell and lower chlorophyll content in immature green fruits [59]. Furthermore, more starch and carotenoids accumulated in the fruits of *SlUVR8*-overexpressing plants [59]. The experiments demonstrated that SlUVR8 plays a vital role in the regulation of chloroplast development in fruits by regulating SlGLK2 and SlHY5. Thus, SlUVR8 is a new candidate to be targeted to enhance both the tolerance to UV-B stress and the nutrient value of fleshy fruits. Therefore, further study is required to demonstrate the detail mechanism of UVR8 on regulating of chloroplast development [59].

## 4. Linkage between Phytohormone and Chloroplast Development in Fleshy Fruits

### 4.1. ABA Signaling Regulates Chloroplast Development in Fleshy Fruits

By screening a tomato mutant pool, a mutant named *high-pigment 3* (*hp3*) with elevated carotenoid content at all stages of fruit development was identified [16]. The mutation in *hp3* corresponds to the gene for zeaxanthin epoxidase (ZEP), a rate-limiting enzyme that converts zeaxanthin to violaxanthin in the pathway for xanthophyll metabolism. The phytohormone abscisic acid (ABA) is derived from xanthophylls, and the *hp3* mutant has 75% lower xanthophyll levels, resulting in ABA-deficiency [16]. Gene expression analysis demonstrated that *FtsZ* (*Filamentous temperature sensitive Z*), which encodes a tubulin-like protein involved in plastid division, was significantly increased in *hp3*, suggesting that plastid division could be elevated in this mutant. Microscopy revealed that the number of plastids per cell of unripe green fruit was increased two-fold in *hp3-1* in comparison to wild-type, and the size of plastids was also larger [16]. Although the ABA signaling pathway appears to be involved in regulating chloroplast development in fleshy fruit, the molecular mechanism by which ABA deficiency affects the expression of *FtsZ* is not known. To further test this hypothesis, two ABA-deficiency mutants, *sitiens* and *flacca*, in which ABA biosynthesis is blocked at the ABA-specific aldehyde oxidase and its molybdenum cofactor, respectively were investigated [16]. They were shown to exhibit higher plastid numbers in unripe green fruit cells. Taken together, ABA signaling takes part in chloroplast development in tomato fruits [16].

The CDD (for COP10, DDB1a and DET1) complex interacts with DET1- AND DDB1-ASSOCIATED1 (DDA1) to form the COP10-DET1-DDB1-DDA1 (CDDD) complex, providing substrate specificity for CULLIN4-RING E3 ubiquitin ligase (CRL4), which is responsible for ABA receptor (PYL) degradation [60]. In addition, in rice, it was found that ABA biosynthesis and ABA signaling are modulated by OsDET1 [61]. It seems that ABA signaling regulates fruit chloroplast development downstream of the CDD complex. However, the mechanism by which ABA signaling regulates fruit chloroplast development is not understood. Since APRR1, a protein related to APRR2, has been reported to interact with ABSCISIC ACID INSENSITIVE 3 (ABI3) [62], it was suggested but not yet demonstrated that APRR2-Like interacts with ABI3 to influence ABA signaling during plastid development and ripening [18].

### 4.2. Auxin Signaling Modulates GLK Expression

Auxin is a well-known phytohormone that modulates plant growth and development mainly through two gene families: the short-lived nuclear protein Aux/IAA family and auxin response factors (ARFs), which regulate auxin-responsive genes on transcriptional level [63]. ARFs can either activate or repress the transcription of auxin-responsive genes by binding to auxin response elements in auxin-responsive gene promoters [63,64]. In *Arabidopsis*, auxin signaling mediated by IAA14 and ARF7/19 has been described to play an inhibitory role in chloroplast differentiation in roots [53]. In tomato, by reverse genetics approaches, *DEVELOPMENTALLY REGULATED 12* (*DR12*) (now named *SlARF4*), one of the *ARF-like* genes, was demonstrated to be a negative regulator of chloroplast development in tomato fruit. The *DR12/SlARF4*-suppressed lines showed a dark-green immature fruit phenotype with increased chlorophyll and the accumulation of starch, glucose and fructose [65]. *SlARF4* expression is high in pericarp tissues of immature fruit; correspondingly, the dark-green phenotype of *SlARF4*-suppressed lines is restricted to fruit [19]. By quantitative RT-PCR, it was found that the expression of *DR12*/*ARF4* was decreased in the fruits of the *hp1* mutant, suggesting that decreased *DR12*/*ARF4* transcription might be one way that plastid compartments are enlarged in immature fruits of *hp1* [66]. Recently, it was reported that SlARF4 binds to the promoter of *SlGLK2* to suppress its transcription activity [67]. In addition, SlARF4 was found to downregulate *SlGLK1* expression in tomato fruits when *SlGLK2* was mutated [19]. In another report, SlARF10, another auxin response repressor, targeted the promoter of *SlGLK1* and positively regulated *SlGLK1* expression in tomato fruits [68]. Overexpression of SlARF10 increased the transcription levels of *SlGLK1*, *SlGLK2*, POR, chlorophyll binding protein 1 (CBP1) and CBP2 in fruit, whereas its downregulation had the opposite effect [68], which suggests SlARF10 is a positive regulator of fruit chloroplast development. Another ARF, SlARF6A directly bound to the promoters of the *SlGLK1*, *CAB* and *RbcS* genes and positively regulated the expression of these genes and *SlGLK2* both in fruits and leaves in tomato [69]. The sizes of chloroplasts were increased in *SlARF6A*-overexpressing plants, and the number of chloroplasts was decreased in *SlARF6A*-downregulated plants. The expression of the *SlARF4* and *AlARF10* genes was independent of *SlARF6A* [69], and the expression of *SBLlARF4* was independent of *SlARF10* [68]. Taken together, these findings show that auxin signaling modulates *GLKs* expression through different ARFs. Besides ARF4, ARF6A and ARF10, whether other ARFs in auxin signaling pathway involved in the transcriptional regulation of chloroplast development in fleshy fruit awaits further study.

### 4.3. Brassinosteroid (BR) Signaling Pathway Modulates GLK Expression

BR is a phytohormone which involved in plant cell expansion and diverse developmental and physiological processes [70,71]. BR signaling has also been demonstrated to regulate fruit pigment accumulation [72]. BRASSINAZOLE RESISTANT 1 (BZR1) is one of two key transcription factors in BR signaling that directly regulate the expression of many target genes [71]. Ectopic expression of *SlBZR1-1D* leads to the setting of fruits with a dark-green shoulder at the mature green stage, a similar phenotype to that of *U* controlled by *SlGLK2*. Indeed, *SlGLK2* expression was upregulated in fruits at the mature green stage when *BZR1-1D* was overexpressed [72]. Previous research revealed that BZR1, phytochrome-interacting factor 4 (PIF4) and ARF6 activate some of the same target genes, and PIF4 and BZR1 facilitate the binding of ARF6 to its target promoters [73]. It is possible that BZR1 regulates the expression of *SlGLK2* through ARF6, as SlARF6A positively regulated the expression of *SlGLK2* both in fruits and leaves [69]. Therefore, BR and auxin are interdependent in regulation of fruit chloroplast development. Furthermore information should be obtain to understand how BR signaling pathway modulates chloroplast development in fruit.

## 5. Working Models for the Transcriptional Regulation of Fruit Chloroplast Development

**Figure 2:**
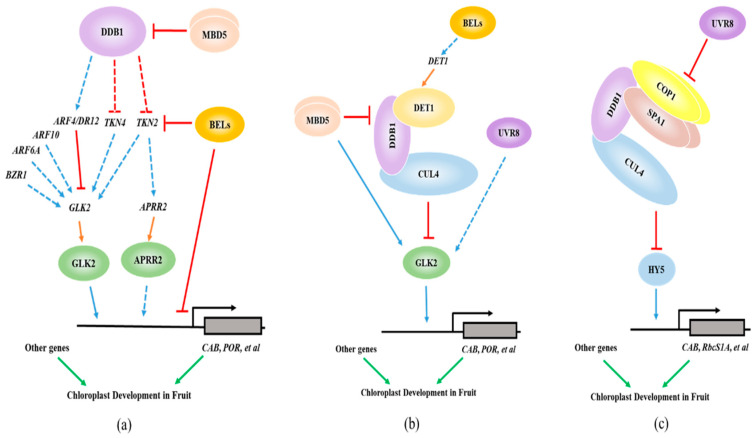
Models for the transcriptional regulation of chloroplast development in unripe fleshy fruit. (**a**) The regulation of *GLK2* and *APRR2* transcription levels to control chloroplast development in fruit. The *KNOX* genes *TKN2* and *TKN4* positively affect *GLK2* and *APRR2* expression to promote chloroplast development. Auxin, via *ARFs*, regulate the expression of *GLK2*, whereas brassinosteroids (BRs), via *BZR1-1D*, promote *GLK2* expression (Liu et al., 2014). *TKN2* regulates the expression of both *GLK2* and *APRR2*, whereas *TKN4* only regulates the expression of *GLK2*. DDB1 acts upstream of *ARF4/DR12*, *TKN4* and *TKN2* to regulate their expression. The MBD5 dimer blocks DDB1 regulating downstream genes. In addition, BEL11 directly represses the transcription of *TKN2*, *CAB* and *POR* by binding their promoters. (**b**) Proteasomal degradation of transcriptional regulator GLK2. The CUL4-DDB1-DET1 ubiquitin ligase complex targets GLK2 for proteasomal degradation. MBD5 inhibits this process by interacting with the subunits of this complex. On the other hand, MBD5 directly interacts with GLK2 to inhibit its degradation. BEL11 enhances the transcript levels of *DET1*, thus helping to form the CUL4-DDB1-DET1 complex. UVR8 also plays a role in accumulating GLK2 protein under UV-B illumination through post-translational regulation. (**c**) Ubiquitination and degradation of transcriptional regulator HY5. HY5 promotes chloroplast development in fruit, although the mechanism is unclear. COP1-SPA1-DDB1-CUL4, an ubiquitin ligase complex that targets HY5 for ubiquitination and degradation, negatively regulates chloroplast development in fruit. UVR8 interacts with the COP1-SPA complex that interferes with the degradation of HY5. Solid blue and red arrows indicate direct regulation, and the dotted arrows represent indirect regulation or the regulation mechanism is uncertain. Solid orange arrows represents gene transcription and translation. Solid green arrows indicate genes that regulate chloroplast development.

In summary, we propose several models for the transcriptional regulation of fruit chloroplast development based on research progress to date (Figure 2). Basically, GLK2, APRR2 and HY5 are master transcription factors that directly regulate the transcription levels of genes related to chloroplast development, photosynthesis and chlorophyll biosynthesis in fruit. At the transcriptional level, *BZR1*, *ARF6*, *ARF10*, *TKN4* and *TKN2* positively regulate *GLK2*, and *TKN2* additionally regulates *APRR2*. ARF4/DR12 negatively regulates the transcription of *GLK2* directly by binding its promoter. In addition, BEL11 and BL4 repress *TKN2*, *CAB*, *POR* and other genes to regulate chloroplast development. DDB1 acts upstream of *ARF4/DR12*, *TKN4* and *TKN2* to regulate their expression, and MBD5 inhibits DDB1′s function.

Additional layers of controls on the transcription factors GLK2 and HY5 occur through post-translational regulation via the CUL4-DDB1-DET1 and COP1-SPA1-DDB1-CUL4 ubiquitin ligase complexes, respectively (Figure 2b,c). MBD5 interacts with the CUL4-DDB1-DET1 complex and inhibits its regulatory function on GLK2. Additionally, MBD5 directly interacts with GLK2 to inhibit its degradation. BEL11 enhances the transcription of *DET1*, thus leading to formation of the CUL4-DDB1-DET1 complex. UVR8 also plays a role in accumulating GLK2 protein under UV-B illumination through post-translational regulation. UVR8 interacts with the COP1-SPA complex(es) that interferes with the degradation of HY5. In addition, LOL1 represses chloroplast development in fruit through uncertain mechanisms. ARF10 directly targets the *GLK1* promoter and transcriptionally activates the expression of *GLK1* in tomato fruit, increasing the chlorophyll content of unripe fruits.

## 6. Improving Fruit Phytonutrients by Regulating Fruit Chloroplast Development

GLK2 is a key transcriptional regulator of chloroplast development and chlorophyll biosynthesis in fruits. In tomato, *GLK2* and *GLK1* overexpression increased chlorophyll levels in green fruit and increased fructose, glucose, soluble solids and lycopene in red fruit [17]. Upregulation of *SlAPRR2-Like* increased the plastid number and area, the content of chlorophyll in green unripe fruits, carotenoids in red ripe fruits [18]. Therefore, GLK2 and APRR2-Like are promising candidates to target for improving fruit phytonutrients. Other approaches also can be exploited to improve our current varieties. For example, for a long time, tomato breeders have selected varieties with uniform light green fruit before ripening, for easier maturity determination, however, these fruits also ripen with reduced sugars and carotenoid, compromising flavor of fresh fruits [17]. Since the point mutation in *SlGLK2* leads to these traits, in order to recover the benefit traits in current varieties, cross them with *SlGLK2* highly expressed tomato germplasms, and subsequently multi generation back-cross can be employed. In another way, the fast developing gene editing systems can be used to edit the mutated *SlGLK2*, thus the function of *SlGLK2* can be restored.

Although tomato *DET1*-impaired mutants (*hp-2*) and constitutively *DET1*-silenced plants showed elevated carotenoids in their mature fruits, severe developmental defects were also observed [31,74]. To avoid the negative effects of *DET1* deficiency on plant growth, one strategy is to specifically repress *DET1* in fruits by using fruit-specific promoters combined with RNAi technology. Using this approach, transgenic plants were found to develop dark-green immature fruits that turned deep red at the mature stage [75]. These deep-red fruits displayed significantly higher contents of carotenoids and flavonoids, whereas other parameters of plant growth and fruit quality were largely unchanged. A similar strategy was employed to specifically repress fruit *DDB1* expression in tomato, resulting in elevated pigment accumulation in fruits without vegetative growth penalty [48]. These studies give us examples of how to employ genes which are disadvantage to plants growth, but possess bright prospect to promoting chloroplast development in fruit.

## 7. Conclusions and Perspectives

Fleshy fruit is the edible part of many plants. The chloroplast in fleshy fruit is not only the photosynthetic organelle for organic carbon synthesis but also the source of chromoplasts for synthesizing special nutrients, such as the carotenoid lycopene (Figure 1). In this review, we have provided a comprehensive overview of the research progress on the transcriptional regulation of chloroplast development in fleshy fruit. Considering the importance of chloroplast development in fleshy fruit and the distinct regulatory mechanisms of chloroplast development in leaves, it is important to identify more players involved in the transcriptional regulation of chloroplast development in fleshy fruit. For example, recently, it was found that *SlELP2L*, an elongator complex protein 2-like gene, regulates chloroplast development and chlorophyll accumulation in tomato fruits through epigenetic modification [76]. Furthermore, negative regulators that promote fruit chloroplast development without yield penalty are eagerly to be identified and investigated, thus they can be the ideal targets to be precisely edited by CRISPR/Cas9 in order to create fruits with improved phytonutrients, and these genes edited plants can be considered transgene free that could easily be applicable.

The following step is to demonstrate the mechanisms by which DDB1 regulates the transcription of *TKN4*, *TKN2* and *ARF4/DR12*, and by which APRR2, HY5, LOL1 and ELP2L regulate fruit-specific chloroplast development need to be clarified. Furthermore, a refined analysis of the relationship between GLK2, APRR2, BEL11, LOL1 and the CDD complex will contribute to more detailed understanding of the mechanism of chloroplast development in fleshy fruit, a mechanism that can be exploited to improve the nutritive value of fruit vegetables and fruits.
